# Multivariate prediction of Saliva Precipitation Index for relating selected chemical parameters of red wines to the sensory perception of astringency

**DOI:** 10.1016/j.crfs.2023.100626

**Published:** 2023-10-26

**Authors:** Cristian Galaz Torres, Arianna Ricci, Giuseppina Paola Parpinello, Angelita Gambuti, Alessandra Rinaldi, Luigi Moio, Luca Rolle, Maria Alessandra Paissoni, Fulvio Mattivi, Daniele Perenzoni, Panagiotis Arapitsas, Matteo Marangon, Christine Mayr Marangon, Davide Slaghenaufi, Maurizio Ugliano, Andrea Versari

**Affiliations:** a Department of Agricultural and Food Sciences, University of Bologna, Italy; b Department of Agricultural Sciences, Division of Vine and Wine Sciences, University of Napoli Federico II, 83100, Avellino, Italy; c Department of Agricultural, Forest and Food Sciences, University of Torino, 10095, Grugliasco, Italy; d Metabolomic Unit, Research and Innovation Centre, Fondazione Edmund Mach, 38010, San Michele all’Adige, Italy; e Department of Wine, Vine and Beverage Sciences, School of Food Science, University of West Attica, Egaleo, 12243, Athens, Greece; f Department of Agronomy, Food, Natural Resources, Animals and Environment (DAFNAE), University of Padova, 35020, Legnaro, Italy; g Department of Biotechnology, University of Verona, via della Pieve 70, San Pietro in Cariano, VR, Italy

**Keywords:** Grape variety, Tannins, Polyphenolic compounds, Chemometric analysis, Sensory analysis

## Abstract

Astringency is an essential sensory attribute of red wine closely related to the saliva precipitation upon contact with the wine. In this study a data matrix of 52 physico-chemical parameters was used to predict the Saliva Precipitation Index (SPI) in 110 Italian mono-varietal red wines using partial least squares regression (PLSr) with variable selection by Variable Importance for Projection (VIP) and the significance of regression coefficients. The final PLSr model, evaluated using a test data set, had 3 components and yielded an R^2^_test_ of 0.630 and an RMSE_test_ of 0.994, with 19 independent variables whose regression coefficients were all significant at *p* < 0.05. Variables selected in the final model according to the decreasing magnitude of their absolute regression coefficient include the following: Procyanidin B1, Epicatechin terminal unit, Total aldehydes, Protein content, Vanillin assay, 520 nm, Polysaccharide content, Epigallocatechin PHL, Tartaric acid, Volatile acidity, Titratable acidity, Catechin terminal unit, Proanthocyanidin assay, pH, Tannin-Fe/Anthocyanin, Buffer capacity, Epigallocatechin PHL gallate, Catechin + epicatechin PHL, and Tannin-Fe. These results can be used to better understand the physico-chemical relationship underlying astringency in red wine.

## Highlights

• Salivary precipitation index (SPI) prediction helps to understand wine astringency.
• Multivariate statistics provided a portable and significant SPI prediction model.
• It reduced from 52 variables to 19, all with significant regression coefficients.
• Procyanidin B1 and Epicatechin terminal unit had the largest regression coefficient.
• PLSr model had: 3 comps., R^2^_test_: 0.630, RMSE_test_: 0.994, and 19 variables (p < 0.05).

## Graphical abstract

**Figure:**
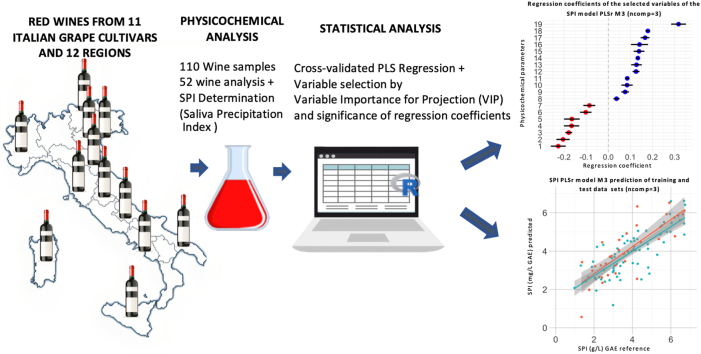

## 1 Introduction

Astringency is one of the most important sensory characteristics of red wine that drives consumer preference (Rinaldi and Moio 2021). Thus, a better understanding of astringency and its prediction is compulsory to produce wine with high consumer liking. One of the first conceptualizations of wine astringency vocabulary defines it as a complex mixture of dryness, roughness, and puckery sensations in the mouth (Lawless et al., 1994). In general terms astringency is due to the loss of lubrication given by saliva, which reduces the friction between oral mucosal surfaces. The main mechanism accepted to explain the perceived astringency in the case of wine is the loss of lubrication due to the precipitation of saliva proteins by wine tannins. The initial molecular binding model was further developed and explained by Jöbstl et al. (2004) in a three-stage interaction process: (i) simultaneous binding of polyphenols to free proteins leading to a compact conformation; (ii) dimerization of them as the concentration of polyphenol increase, and (iii) precipitation as a consequence of large aggregate particles. Although precipitation of salivary proteins, mainly proline-rich proteins, is one of the most accepted mechanisms to explain astringency phenomena, further mechanisms were proposed including (i) the activation of the mechanoreceptors, (ii) the interaction with the oral mucosa by the tannin-protein precipitates, or (iii) the direct interaction of tannins with salivary proteins (Rinaldi and Moio, 2021; González-Muñoz et al., 2022).

Tannins are among the most important polyphenolic compounds in red wine and they could be divided into two main groups: (i) condensed tannins or proanthocyanidins, which are polymeric flavan-3-ols, consisting in varying degrees of polymerization of (+)-catechin and (−)-epicatechin monomer units which can, in turn, be esterified with gallic acid; (ii) hydrolyzable tannins, which have a central core of monosaccharides that are esterified with gallic or ellagic acids or are oligomers of them, known as gallotannins and ellagitannins. Although the latter has been reported to give a greater sensation of astringency than condensed tannins at equal molar concentration, with ellagitannins being often more astringent than gallotannins, the hydrolyzable tannins usually play a minor role in the perceived astringency due to their low concentration in wine (Soares et al., 2017). Concerning condensed tannins, it has been reported that their interaction with proteins depends on their concentration in the wine, molecular weight/mean degree of polymerization (mDP), interflavanic bond type, galloylation (galloyl groups), hydroxylation pattern of B-ring, and stereochemistry conformation (Strati et al., 2021; González-Muñoz et al., 2022). Nevertheless, due to the structural rearrangements due to both the hydrolysis and the polymerizations of tannins during wine aging (including their oxidation), the enhancement in astringency seems not linear to the increase in the number of hydrophobic functional groups (Ma et al., 2014).

Within flavonoids, in addition to flavan-3-ols, anthocyanins may also play an important role in protein precipitation, although their role in the perception of wine astringency is not yet fully understood. In a study of Paissoni et al. (2018) anthocyanins were observed to react with both BSA and salivary proteins in model solution, although the interaction with salivary protein was stronger and reacted more actively with cinnamoylated anthocyanins. A further study (Paissoni et al., 2020) showed that the presence of anthocyanins can modify the intensity of the astringency sensation and its sub-qualities, due to their interaction with other polyphenols. It was shown that the addition of anthocyanin glycoside to the seed extract was perceived as more astringent with a harsher sub-quality. However, the opposite effect occurred when it was added to skin extracts, where it was perceived as a lower surface smoothness sub-quality, although the intensity of the overall astringency did not vary. Anthocyanins can also modify astringency through modification of tannin structure. In this sense, the incorporation of anthocyanins into proanthocyanidins has shown a greater effect on the attenuation of astringency, compared to the intensification of astringency due to the increase in the degree of polymerization (Weilack et al., 2021).

Other wine constituents have also been postulated as responsible for modulating astringency, among them: ethanol, organic acids, pH, cations, as well as polysaccharides; with a multi-component nature of astringency perception, where the correlation between wine parameters and perceived astringency is not entirely clear at the moment (Sáenz-Navajas et al., 2019; González-Muñoz et al., 2022). Several efforts have been made to correlate the physico-chemical parameters of wine with its perceived astringency, using different assays, as follows: bovine serum albumin (BSA) protein precipitation assay (R^2^ = 0.82), phloroglucinolysis (R^2^ = 0.73), gel permeation chromatography (R^2^ = 0.74) (Kennedy et al., 2006); methylcellulose protein precipitation MCP (R^2^ = 0.83) (Mercurio and Smith, 2008); saliva precipitation index (SPI) (R^2^ = 0.97) (Rinaldi et al., 2012a, Rinaldi et al., 2012b), and absorbance at 230 nm (R^2^ = 0.705) (Boulet et al., 2016). Piombino et al. (2020) tried to correlate astringency sub-qualities with the total phenol concentration by Folin-Ciocalteu assay and proanthocyanidins concentration by warm acid hydrolysis assay with ferrous salt as catalyst. They found a significant positive correlation for dryness (r = 0.56 and r = 0.71, respectively) and harsh (r = 0.48 and r = 0.47).

The Saliva Precipitation Index (SPI) is an advanced assay based on SDS-PAGE electrophoresis that measures the binding and precipitation of salivary proteins towards wine polyphenols with the attempt to predict the perceived astringency of wines (Rinaldi et al., 2010). The method involves the selection of bands that best correlate with the perceived astringency, and the calibration curve over a wide range of concentration (0.1–5.0 g/L) allows the determination of SPI expressed in gallic acid equivalent (g/L). Parameters such as saliva-to-wine ratio, saliva typology, and temperature of binding reaction have been optimized in this methodology to reach a high correlation with perceived astringency of R^2^ = 0.97 (Rinaldi et al., 2012a, Rinaldi et al., 2012b). In an effort to simplify the application of the above method, Qi et al. (2023) have used the response surface methodology (RSM) to develop an artificial saliva by which to perform an artificial saliva precipitation index (ASPI) that optimizes a higher coefficient of determination value of prediction (R^2^_pred_) through the mixture of different proteins with different concentrations.

Although several studies have demonstrated the ability to predict astringency in wine, the overall relationship between the many physico-chemical variables and perceived astringency is still a challenge. Thus, multivariate analysis is considered a valuable approach to further model the relationship between astringency and chemical parameters. In this view, preliminary studies focused either on multiple linear regression (R^2^ = 0.909) (Boulet et al., 2016), or non-linear PLS regression (RMSE = 0.19) (Sáenz-Navajas et al., 2019). The multivariate approach could aid winemakers in gaining a holistic understanding of the factors that contribute to wine quality and in developing precision practices during the various stages of the winemaking process, from vine to wines, the latter with desirable sensory qualities.

The aim of this study is to explore the feasibility of the prediction of SPI by using a multivariate approach to unravel the most important variables in predicting red wine astringency. This was performed using different Italian mono-varietal red wines, using partial least squares regression (PLSr) prediction tool to train models with a train data set, with randomized and replicate k-fold cross validation, variable selection through the Variable Important for Projection (VIP) scores, the significance of regression coefficients, and a randomized test to generate a statistically significant predictive model for SPI, that was finally evaluated with a test data set.

## 2 Materials and methods

### 2.1 Wines

The study was performed with mono-varietal commercial red wines from eleven grape varieties harvested and vinified in the 2016 vintage from twelve regions of Italy and collected directly from local wineries in early 2017. The wines included: Aglianico from Campania (AGL, n = 10); Cannonau from Sardinia (CAN, n = 9); Corvina from Veneto (COR, n = 7); Montepulciano from Abruzzo (MON, n = 9); Nebbiolo from Piedmont (NEB, n = 11); Nerello Mascalese from Sicily (NER, n = 3); Primitivo from Puglia (PRI, n = 11); Raboso del Piave from Veneto (RAB, n = 10); Sagrantino from Umbria (SAG, n = 10); Sangiovese from Romagna (SAR, n = 12); Sangiovese from Tuscany (SAT, n = 7) and Teroldego from Trentino (TER, n = 11). Overall, the dataset included 110 red wines analyzed for 52 physico-chemical parameters, and Table 1 shows the distribution of the number of samples per wine grape variety and origin. Specifications for winemaking protocol and sample storage, including wine analysis methodologies and the metadata of samples, are fully available in the literature from our previous works (Arapitsas et al., 2021; Giacosa et al., 2021; Marangon et al., 2022), and are listed in Supplementary Table S1 together with the article where the method is presented.

**Table 1:** Number of samples distribution per wine grape variety and origin.

Code	Original data set	Outliers	Training data set	Test data set
AGL	10	1	6	3
CAN	9	–	6	3
COR	7	1	4	2
MON	9	1	5	3
NEB	11	1	7	3
NER	3	–	2	1
PRI	11	1	7	3
RAB	10	–	7	3
SAG	10	–	7	3
SAR	12	–	8	4
SAT	7	–	5	2
TER	11	–	8	3
Total	110	5	72	33

### 2.2 SPI analysis

The SPI analysis was carried out in triplicate as already described Rinaldi et al. (2014). The commercial Experion Pro260 analysis kit and the Experion system were used for the SPI determination. The saliva samples were analyzed before and after the binding reaction with wine tannins under controlled conditions. The SPI was calculated by the percentage reduction of the fluorescence signal of salivary proteins compared to control saliva. Results are expressed as gallic acid equivalent (mg/L GAE).

### 2.3 Statistical procedure and methodology for multivariate prediction of SPI

**Fig. 1:**
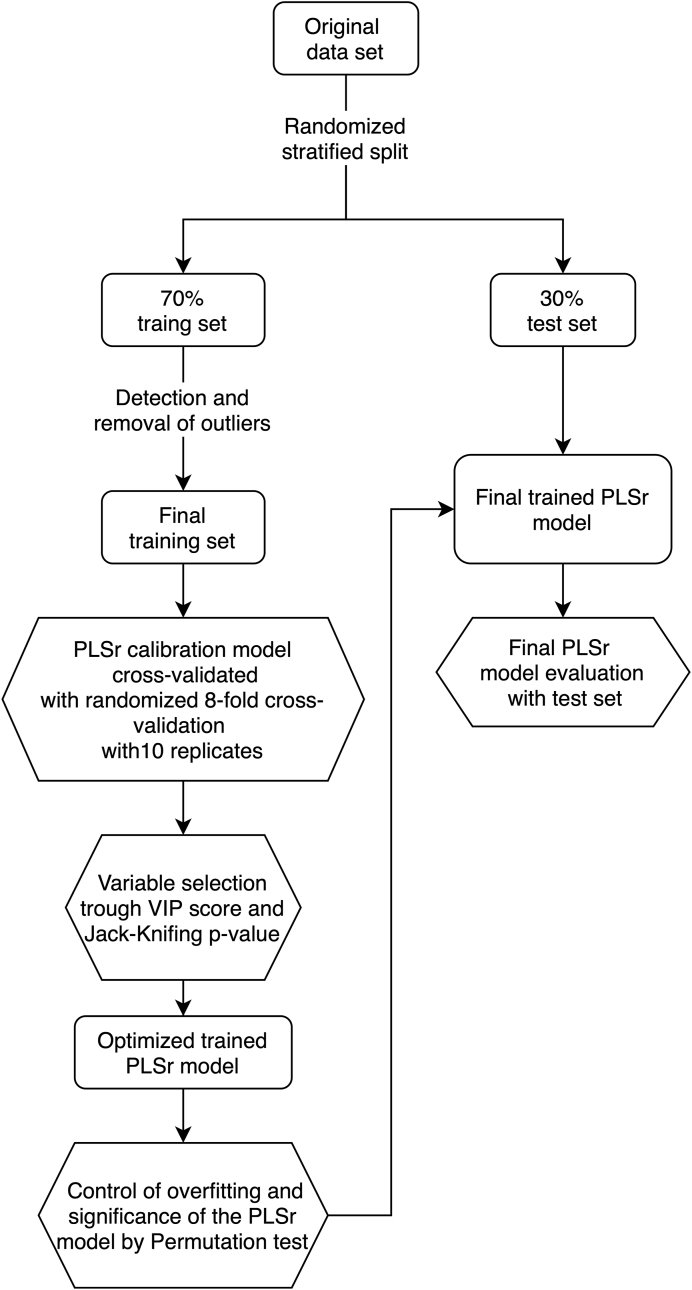
Scheme of PLSr model training, cross-validation, and test for SPI.

As shown in Fig. 1, the statistical process began with a random, stratified division of the raw data set by wine grape variety and production region into training (70%) and test (30%) data sets. This means that the representative percentage of the original data set for each wine grape variety and region was retained as much as possible in the train and test data sets. Outliers in the training data set were then detected using Cook's distance (Cook, 1977), and five of them were removed from the training data set, leaving the test data set intact until the final model evaluation. Then, the “pls” function of the “mdatool” package of R-Studio® (Kucheryavskiy, 2020) was used to auto-scale and center the training data set and generate PLSr models, which were cross-validated using a random 8-fold cross-validation with 10 repetitions on the entire training dataset. The selection of folds and replicates was chosen as a good trade-off to allow an efficient cross-validation of the model and calculate the significance of the regression coefficients for the independent variables with a sufficient degree of freedom for jack-knifed p-value in the t-distribution. However, preliminary evaluations were performed (not shown), and the results obtained were the same as with 4-fold and 10- fold. Therefore 8 folds were chosen for the reason mentioned above. From this training procedure, the different statistical parameters of the models were RMSE_cal_ (root mean squared errors of calibration), RMSE_cv_ (root mean squared errors of cross-validation), with their confidence intervals and p-values through Jack-Knife method, and the VIP scores for each independent variable of the model (physicochemical parameters). Subsequently, an optimized PLSr training model was obtained by excluding independent variables with a VIP score less than 1.0 and a significant p-value of the regression coefficient greater than 0.1. The resulting model had an improved RMSE_cv_, in which the *p*-value of the regression coefficient for each independent variable was re-calculated, and all those with a *p*-value >0.05 was removed. Thus, an improved PLSr model was obtained with 19 independent variables with significant regression coefficients for all of them.

The VIP score is an indicator of the combined contribution of the independent (Xs) and dependent (Y) variables. Which, by including the weights of the PLSr model, is able to express the covariance between the independent and dependent variables. Thus, the accuracy of the description of the dependent variable and the relevance for the model of the independent variables of this information are evaluated similarly(Andersen and Bro, 2010). On the other hand, the Jack-Knife method consists of calculating the uncertainty of the estimation of a specific statistical parameter, generally the regression coefficient, and calculating its *p*-values and confidence interval for each independent variable by cross-validating the model through data resampling and thus obtaining results from different conformal models that are used for the calculations (Andersen and Bro, 2010). After variable selection, a randomized test was performed to evaluate the significance of the optimized PLSr model. The optimal number of components was selected to generate a significant model in which an overfit check was performed to obtain the final significant and not overfitted trained model.

The randomized test is valuable when data pre-processing has been performed, which is used to reject uninformative components for the PLSr model, similar to the *p*-value used in the statistical test. It takes the covariance between the dependent (X) and independent (Y) variable scores to calculate a t-statistic. This calculation is repeated to obtain a histogram of the null distribution, i.e., the distribution of the statistic over the randomly permuted values of the independent variable. The alpha parameter was then calculated by the number of times an equal or higher t-statistic is obtained compared to the unswapped y-values. Thus, the non-significant components will have a lower covariance of the permuted Y, compared to the non-permuted ones, hence a higher alpha value. Which, if greater than the critical value (5–10%), is considered to generate non-significant models (Wiklund et al., 2007).

On the other hand, overfitting control consists of avoiding models that violate parsimony. That is, rejecting the use of models with unnecessary terms or more complicated approaches that may lead to not achieving the corresponding benefit or to underperforming compared to a simpler model (Hawkins, 2004). In this respect, separating the data matrix into training and test data sets at the beginning of the statistical process is a usual first direct measure to avoid overfitting, as is done in this work. But, operations such as the removal of outliers or taking different PLS model configurations may result in model overfitting (Shmueli et al., 2016).

Therefore, it is essential to implement additional actions in the final model inspection to ensure that the models are not over-fitted. One option is the control of the “|R^2^_cal_ − R^2^_cv_| vs. R^2^_cv_” plot (R^2^_cal_: the determination coefficient of calibration, and R^2^_cv_: the determination coefficient of cross-validation). Where |R^2^_cal_− R^2^_cv_| should not be more than 20%, and the optimal Pareto solution (maximal number of components for an optimal model) should be placed at the inflection point of the outer convex hull in the plot (Mendez et al., 2019). Another interesting option is the control of the plot “RMSE_cv_/RMSE_cal_ ratio vs. RMSE_cv_” (being RMSEcv: the root mean square error of cross-validation, and RMSE_cal_: the root mean square error of calibration), which provides complementary information to the previous one, but unlike the coefficients of determination (R^2^_cal_ or R^2^_cv_), the RMSE_cal_ and RMSE_cv_ are expressed in the same units as those being predicted for the model (Kucheryavskiy, 2020).

The final step in the process involved the evaluation of the predictive ability of the optimized PLSr model using the test data set, which was separated and untouched from the beginning of the process. This evaluation included obtaining the test root mean square error (RMSE_test_), the test coefficient of determination (R^2^_test_), and the *p*-value of the regression coefficient of the previously selected dependent variables, which helped to determine the effectiveness and reliability of the final model.

## 3 Results

### 3.1 SPI of red wines

Table 2 shows the minimum, maximum and mean SPI values for each mono-varietal Italian red wines. As expected, the range of SPI for each cultivar was large as observed already for astringency among the same wines (Piombino et al., 2020). Also, basic wine parameters showed high inter-varietal diversity as previously reported (Giacosa et al., 2021).

**Table 2:** SPI (g/L of gallic acid equivalent) after outlier remotion.

Code	SPI Training data set				SPI Test data set			
	Min	Max	Mean	sd	Min	Max	Mean	sd
AGL	4.7	7.3	5.9	1.0	4.3	7.0	5.9	1.5
CAN	2.6	4.8	4.0	0.9	2.7	6.7	4.6	2.0
COR	1.0	2.4	1.7	0.6	1.3	2.4	1.9	0.8
MON	2.3	4.9	3.6	1.0	2.9	5.4	4.0	1.3
NEB	2.2	6.7	4.5	1.7	2.4	6.2	4.0	2.0
NER	1.3	2.1	1.7	0.6	2.1	2.1	2.1	–
PRI	1.7	4.2	3.2	0.8	1.4	3.1	2.1	0.9
RAB	3.7	6.2	5.1	1.1	3.7	6.6	4.8	1.6
SAG	4.3	6.7	5.8	0.9	4.4	5.9	5.4	0.8
SAR	1.9	5.3	3.5	1.1	2.4	4.0	3.1	0.7
SAT	3.1	3.8	3.4	0.3	2.4	3.6	3.0	0.8
TER	1.9	3.4	2.9	0.5	1.9	3.1	2.6	0.6
Total_mean	2.5	4.8	3.8	0.9	2.7	4.7	3.6	1.2
Total sd	1.1	1.6	1.3	0.4	1.0	1.7	1.3	0.5

### 3.2 SPI PLSr model

As can be seen in the summary of Table 3, the raw training data set was analyzed with the SPI PLSr model M0 with 77 samples and 1 component that obtained an R^2^_cal_ of 0.298, with an RMSE_cal_ of 1.276, and an R^2^_cv_ of 0.207 with an RMSE_cv_ of 1.357. Then, five outliers were removed (one sample of MON, COR, AGL, PRI and NEB respectively), obtaining the SPI PLSr model M1 with 72 samples and 5 components that gave an R^2^_cal_ of 0.713, with an RMSE_cal_ of 0.808, and an R^2^_cv_ of 0.290 with an RMSE_cv_ of 1.271.

**Table 3:** Summary results of the training and evaluation process for the SPI PLSr model.

Model	Treatment^a^	Nº samples	Nº independent variables	PLS components	Statistical parameters of training process				Statistical parameters for model evaluation	
					R^2^_cal_	R^2^_cv_	RMSE_cal_	RMSE_cv_	R^2^_test_	RMSE_test_
m0	Non	77	52	1	0.298	0.207	1.276	1.357	0.175	1.485
m1	OR	72	52	5	0.713	0.290	0.808	1.271	0.436	1.227
m2	VS	72	19	4	0.652	0.452	0.890	1.117	0.607	1.025
m3	PT&OC	72	19	3	0.624	0.444	0.925	1.125	0.630	0.994

a Treatment codes: Non: without treatment, OR: Outlier removal, VS: Variable selection, and PT&OC: Permutation test and overfitting control. The rest of the codes are explained in the text.

After removal of outliers, the VIP scores of each independent variable and the *p*-values of their regression coefficients were calculated. Variables with VIP scores >1.0 and *p*-values <0.1 were eliminated, leaving 20 independent variables. Next, independent variables with non-significant regression coefficients (with a *p*-value of regression coefficients >0.05) were removed until only the ones with significant regression coefficients remained in the model. In this step, just one independent variable was eliminated, resulting in a final selection of 19 independent variables. The process was completed by generating a new M2-optimized SPI PLSr model with 4 components based on the selected variables. This model returned an R^2^_cal_ of 0.652 with an RMSE_cal_ of 0.890 and an R^2^_cv_ of 0.452 with an RMSE_cv_ of 1.117, indicating an improvement in performance.

### 3.3 Evaluation of the PLSr model

**Fig. 2:**
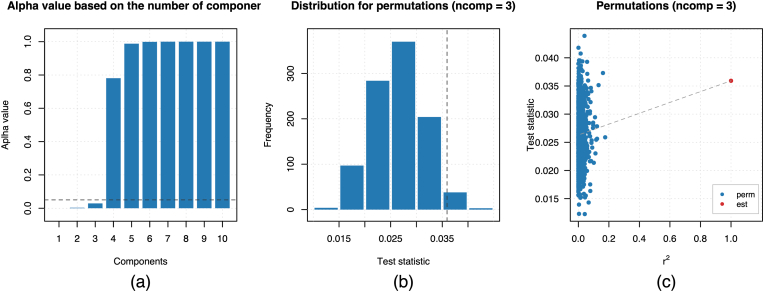
Statistics of Randomized Test with 1000 permutations for the final SPI trained PLSr model. Legend: (a) Alpha values vs. PLSr components, (b) T-statistic permutation histogram, and (C) Correlation between permuted and original Y.

Subsequently, a randomized test was performed on the SPI PLSr model M2 to select the optimal number of components that generate a significant model, suggesting three components with an alpha-value of 0.029, as seen in Fig. 2.

**Fig. 3:**
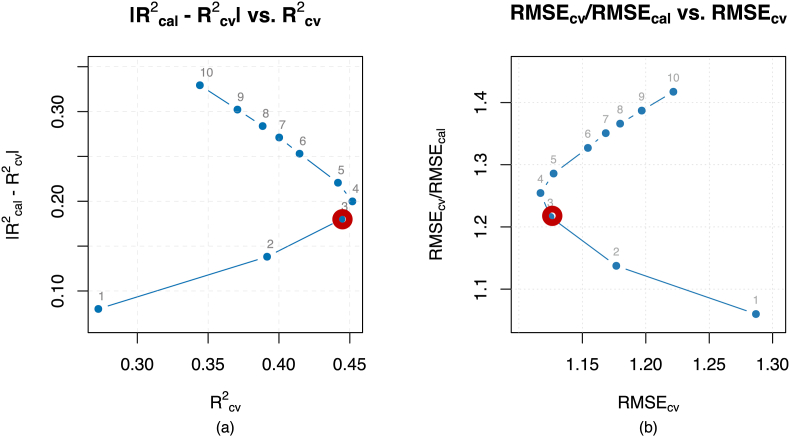
SPI PLSr model overfitting inspection. Legend: (a) Determination coefficient of calibration (R^2^cal) minus determination coefficient of cross-validation (R2cv) vs. determination coefficient of cross-validation (R^2^cv). (b) Root mean square error of cross-validation (RMSECV) divided by root mean square error of calibration (RMSEcal) vs. root mean square error of cross-validation (RMSECV).

After this process, overfitting was checked, as shown in Fig. 3 to confirm that the number of components selected would not generate an overfitted model. In this respect, no signs of overfitting were found since in the graph "|R^2^_cal_-R^2^_cv_| vs. R^2^_cv_” the three components previously selected are below the 20% threshold of |R^2^_cal_-R^2^_cv_| and under the inflection point of the outer convex hull (Mendez et al., 2019). Neither are there any signs of overfitting when analyzing the graph of the “RMSE_cv_/RMSE_cal_ ratio vs. RMSE_cv_”. Although, from the point of view of Pareto optimization in both cases 4 components are the optimal number of components, as also indicated above in the SPI PLSr model M2, after a randomized test, it was found that from four components onwards, non-significant models are generated. Therefore, although three components are not the optimum solution, it is the maximum number of eligible PLSr components that do not show any presence of overfitting but generate a significant PLSr model. Ultimately, it was trained the final and significant SPI PLSr model M3 with 72 samples, 19 independent variables, and three components that achieved an R^2^_cal_ of 0.624 and RMSE_cal_ of 0.925.

**Fig. 4:**
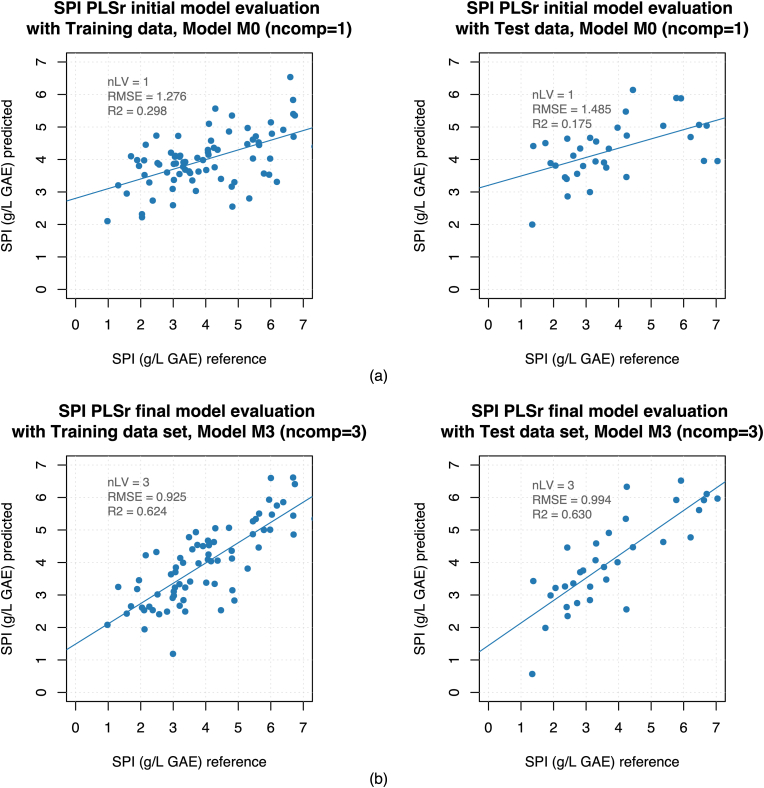
PLSr Linear regression plots of: (a) initial PLSr model vs. (b) final PLSr model evaluation.

Afterward, when the final SPI PLSr model M3 was evaluated with the test data set, it obtained an R^2^_test_ of 0.630 and an RMSE_test_ of 0.994. The plots of the PLS linear regression and their statistical parameters for evaluation results of the SPI PLSr model M3 can be seen and compared with the initial starting point of model M0 in Fig. 4, where a substantial improvement in the predictive ability of the M3 model evaluation is observed.

**Fig. 5:**
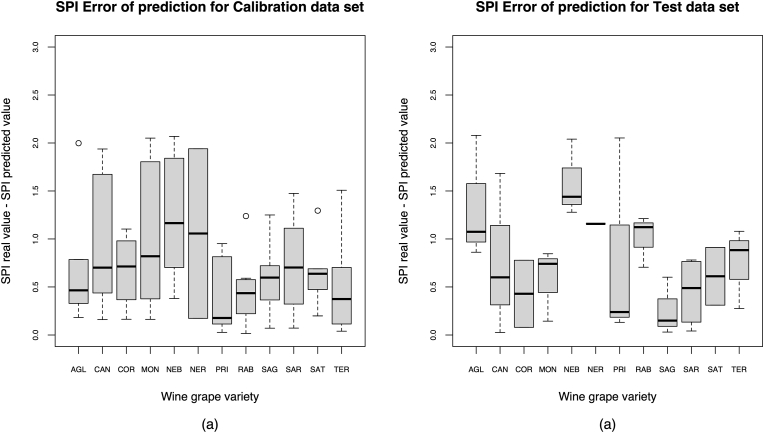
SPI prediction errors (g/L of GAE) of final SPI PLSr model M3. Legend: (a) using the training data set (b) using the test data set.

Fig. 5 shows the final performance of the SPI PLSr M3 model in terms of RMSE prediction error by wine grape variety and region of origin, for the training data set and the test data set. It can be observed that the lowest prediction error is presented by SAG, COR, SAR, and SAT respectively, while the highest errors of prediction correspond to NEB and AGL, with a very large dispersion in the case of CAN and PRI. NER on the other hand, presented only one sample due to its low number of samples in the original data set (3).

**Fig. 6:**
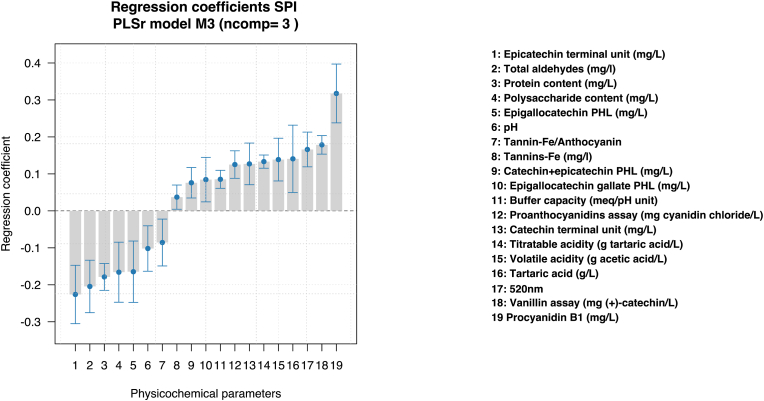
Regression coefficients of independent variables (physicochemical parameters) with its confidence intervals of PLSr model M3. Regression coefficients and confidence intervals are auto-scaled and centered.

Fig. 6 shows a graph with the regression coefficients of all the dependent variables finally selected that were also significant (*p*-value<0.05), together with their confidence intervals, of the final SPI PLSr M3 model. It can be seen that the significant variables found for the model are ordered in decreasing order according to their absolute magnitude of the regression coefficient as follows: Procyanidin B1 (mg/L), Epicatechin terminal unit (mg/L), Total aldehydes (mg/L), Protein content (mg/L), Vanillin assay (mg (+)-catechin/L), absorbance at 520 nm, Polysaccharide content (mg/L), Epigallocatechin PHL (mg/L), Tartaric acid (g/L), Volatile acidity (g acetic acid/L), Titratable acidity (g tartaric acid/L), Catechin terminal unit (mg/L), Proanthocyanidin assay (mg cyanidin chloride/L), pH, Tannin-Fe/Anthocyanin, Buffer capacity (meq/pH unit), Epigallocatechin gallate PHL (mg/L), Catechin + epicatechin PHL (mg/L) and Tannin-Fe (mg/L).

### 3.4 Portability of the model

The next equation (1) is the resulting linear PLS regression function for final SPI PLSr M3 model with its raw regression coefficients. Therefore, it is possible to directly add the values of the variables without scaling or centering.

(1) SPI (g/L GAE) = 2.4385 + a * 1.5492 + b * 0.3903 + c * 0.1844 + d * 0.1102 + e * 0.0329 + f * 0.0145 + g * 0.0068 + h * 0.002 + i * 0.0004 + j * 0.0003 + k * 0.0002 + l * 0.0001 + m * −0.0016 + n * −0.0017 + o * −0.0101 + p * −0.0101 + q * −0.0221 + r * −0.0337 + s * −0.7919

Where:

a: Volatile acidity (g acetic acid/L)

b: Tartaric acid (g/L)

c: Titratable acidity (g tartaric acid/L)

d: absorbance at 520 nm

e: Procyanidin B1 (mg/L)

f: Buffer capacity (meq/pH unit)

g: Catechin terminal unit (mg/L)

h: Epigallocatechin gallate PHL (mg/L)

i: Vanillin assay (mg (+)-catechin/L)

j: Catechin + epicatechin PHL (mg/L)

k: Proanthocyanidins assay (mg cyanidin chloride/L)

l: Tannins-Fe (mg/l)

m: Epigallocatechin PHL (mg/L)

n: Polysaccharide content (mg/L)

o: Total aldehydes (mg/l)

p: Protein content (mg/L)

q: Epicatechin terminal unit (mg/L)

r: Tannin-Fe/Anthocyanin

s: pH.

## 4 Discussion

In general terms, procyanidins have been related to some degree to human salivary precipitation in different publications according to their conformational structure and polymerization. For example, Sun et al. (2013), proved that both saliva precipitation and astringency sensation depend on the degree of polymerization of procyanidins, being greater as it increases (polymers > oligomers), with the best fitting (R^2^ = 0.98) at 0.86–1.2 g/L of procyanidins. Ma et al. (2016) confirmed an increased reactivity of salivary proteins with non-galloylated procyanidins from wine based on their degree of polymerization (up to five) as follows: monomers < dimers < trimers < tetramers, with little effect for the monomers, and no pentamers found after the protein reaction.

Procyanidin B1 concentration in wines remains the most important variable for the purposes of the final SPI PLSr model M3, with a relatively high significance and positive regression coefficient for the model. A tentative explanation for the high positive magnitude of the regression coefficient of procyanidin B1, as seen in Fig. 6, lies in the spatial configuration of it: epicatechin-(4β→8)-catechin, having a catechin as its terminal unit. This is because the colloidal behavior of proanthocyanidins would change mainly according to their 3D configuration and consequently their interaction with saliva proteins (Dufourc 2021). However, it is important not to confuse a higher regression coefficient of an independent variable with a cause-effect relationship, it only indicates that it has a greater influence on the prediction of the dependent variable than other independent variables. In this regard, one explanation for this result may lie in the importance of the procyanidin B1 oligomer in grapes and wine as fingerprint tool for a compositional-related issue, together with other parameters selected for the SPI PLSr model M3.

Regarding flavan-3-ols, epicatechin was associated with an increase in astringency in contrast to catechin at the same concentration (Thorngate and Noble, 1995), whereas in another study catechin showed higher salivary protein precipitation compared with epicatechin at the same concentration (Kallithraka et al., 2001). Sáenz-Navajas et al. (2019) hypothesized that different non-linear trends in astringency response would be related to the procyanidins content (%PC), calculated as the sum of catechin and epicatechin subunits and terminal units divided to the total tannin concentration, and the ratio of the structural epicatechin/catechin subunit. The astringency prediction showed a sharp negative response when the proportion of procyanidins was <68% (thus decreasing astringency with increasing epicatechin/catechin ratio), being positive between 68 and 76% and null with a PC >76%.

In our work, the variable positively related to the increase of SPI is the sum concentration of structural monomers catechin PHL and epicatechin PHL. The catechin terminal unit (58%) had a positive influence on the prediction of SPI, in contrast to the epicatechin terminal unit (29%). The terminal unit of procyanidin B1, which obtained the highest positive regression coefficient, is in fact catechin. All this suggests that it is not the concentration of free flavan-3-ols, but the concentration and overall structure of procyanidins the important one for the prediction of SPI, as well as the structure of their structural monomers, as was observed with epigallocatechin PHL and epigallocatechin gallate PHL, where the concentration of epigallocatechin PHL showed a positive relationship with SPI, while the concentration of epigallocatechin PHL gallate exhibited a negative influence on the prediction of SPI, reaffirming the importance of the global and internal structure of procyanidins for the prediction of SPI.

It is well known that the vanillin assay measures both the monomeric flavanols and the low molecular weight flavan-3-ols polymers, with specificity for catechin, and its value is affected by polymerization with a strong decrease with wine aging (Sarkar and Howarth, 1976). In contrast, the proanthocyanidin assay measures specifically high molecular weight flavan-3-ols polymers (more than 5 units) and it is not able to measure hydrolysable tannins (Gambuti et al., 2020; Giacosa et al., 2021). In this regard, the similarity of regression coefficient between proanthocyanidins and vanillin assays for the final PLSr M3 model suggests that high (proanthocyanidins assay) and low molecular weight proanthocyanidins (vanillin assay) play a similar role for prediction of SPI.

In addition to the above, a preliminary sensory study carried out on the same sample set of mono-varietal Italian red wines found that total proanthocyanidins were significantly correlated with diverse sub-qualities of astringency, mainly to the drying sensation (r = 0.708) (Piombino et al., 2020).

Concerning the negative influence of Tannin-Fe/Anthocyanin ratio on SPI prediction, it could be explained considering that total anthocyanins (sum of free and polymeric pigments) are used to determine this ratio; thus, lower Tannin-Fe/Anthocyanin values could be related to higher values of denominator which included free anthocyanins and pigmented polymers. Higher values of total pigments due to the inclusion of anthocyanins in small tannin oligomers could partly explain the decrease in astringency that could partly explain the decrease in astringency as it occurs as the wine ages, along with tannin cleavage reactions (Cheynier et al., 2006). In this sense, this result could be understood by presuming a tannin-Fe/anthocyanin ratio relation with SPP (small polymeric pigments) and LPP (large polymeric pigments) and its effect on protein precipitation and wine astringency. LPP has been shown to correlate with the intensity of astringency in young wines (Rinaldi and Moio, 2018; Rinaldi et al., 2020a). However, also a decrease in SPI during five years of bottle aging of red wines was already observed (Gambuti et al., 2020) and, when wines age, the formation of pigmented polymers due to oxidation correlates with the perception of velvety sub-quality (Rinaldi et al., 2020b, 2021). Therefore, the higher the total anthocyanins value (which included monomeric and polymeric anthocyanins), the higher the SPP and LPP values were. However, it is important to clarify that these results should be better understood in the future, since SPP and LPP were not selected as variables that optimize SPI prediction in this study, but they were associated with Tannin-Fe/Anthocyanin ratio by PCA analysis (positively related with SPP and negatively with LPP) in a previous work with the same data set (Giacosa et al., 2021).

Total aldehydes concentration showed a negative influence on the prediction of SPI most probably as indirect consequence of the oxidation of alcohols by the hydroxyl radical during the vinification and aging process. In this regard, acetaldehyde may have played an important role, as it bridges with flavanols in a series of well-documented reactions, generating “ethyl-linked” products, ultimately leading to reduction of astringency by polymerization or precipitation of flavonoids (Waterhouse and Laurie, 2006).

With respect to polysaccharides and protein concentration. It is known that grape and yeast polysaccharides tend to inhibit or stabilize salivary protein precipitation at the concentration that they can be found in wine, depending on the polysaccharide and tannin structure (Weilack et al., 2023), as well as the type of saliva and presence of salts (Manjón et al., 2021). Which agrees with the work of Pascotto et al. (2021) and adds that the molecular weight of polysaccharides is positively related to the astringency sub-quality of smoothness. However, the effect of polysaccharides and proteins explained in these studies could also be understood through the work of Bindon et al. (2016), who studied the size of macromolecular complexes in wine by nanotracking analysis (NTA) after microwave maceration. They found that a small increase in polysaccharides and proteins allows a further increase in tannin concentration without an imbalance in the system of stable tannin-polysaccharides (mannoproteins) complexes, increasing their concentration but not their size. The negative influence found in this work between polysaccharide content and SPI prediction is also in agreement with a previous work in which mannoproteins were able to reduce the precipitation of salivary proteins estimated by means of the SPI assay (Rinaldi et al., 2012a, Rinaldi et al., 2012b).

In addition, the effect of wine protein content on salivary protein precipitation has been little to any studied in red wine. Recently it has been found that the proteins are present in greater quantities in red wine than previously thought. Different varieties of *Vitis vinifera* spp. have reported a range of 38–58 mg/L and 120–381 mg/L in the case of *Vitis* spp. *Hybrids* (Kassara et al., 2022). In our study, after outlier removal, the wines showed a protein concentration range from 3.9 to 118.2 mg/L, with a mean of 43.18 mg/L. Interestingly, there is some evidence of the interaction between dimeric procyanidins B1 and B2 with grape proteins, however it is not yet clear how it could behave with more polymerized tannins (Di Gaspero et al., 2020). Thus, if wine proteins interact with wine tannins, they could be negatively related to astringency due to a possible competition with salivary proteins by the same interaction with tannins.

Further variables investigated includes organic acids, pH and buffer capacity. The prediction of SPI increase with the increase of total and volatile acidity, and tartaric acid. These results are partly in agreement with the work of Kallithraka et al. (1997) whom proposed that acidity by itself could have a precipitating action on tannin-protein complexes already formed in the mouth, thus increasing their astringency. Actual studies in which the effect of pH (Gambuti et al., 2022) and organic acids (Picariello et al., 2019) on SPI was determined confirmed this hypothesis. In addition, the effect on the astringency of oligomeric tannins (i.e., procyanidins) has been tested in model wine by Fontoin et al. (2008), obtaining a decrease of astringency with increasing of the pH but with no response for tartaric acid at constant pH. On the other hand, it was observed that volatile acidity and total acidity concentration showed a positive linear relationship with the increase in perceived astringency, which became more evident as the concentration of them increased (Sáenz-Navajas et al., 2019).

The general effect and the mechanism of action where the acidity pH affects astringency has been recently explained. Zhao et al. (2023a) found that pH, and not titratable acidity, showed the best correlation with the intensity of astringency (r = −0.70), its duration (r = −0.82) and the sub-qualities of dryness (r = −0.69) and pucker (r = −0.82). Regarding the mechanism of action, the same research group (Zhao et al., 2023b) proposed that in first instance tartaric acid is able to change the hydrogen and hydrophobic bonds in the protein-polyphenol compounds, and thus stretch the protein structure due the formation of ternary complexes of tartaric acid. Although, this behavior was found up to 3 g/L of tartaric acid concentration, but then its effect is masked. The second mechanism is through its impact on pH, where it was found that a lower in pH increases the fluidity of the saliva layer. This last effect was independent of tartaric acid concentration, thus increasing it concentration but maintaining the pH does not increment the fluidity of the saliva layer, as well as the effect found in the same work for SPI where its increase was also related to pH and not to the concentration of tartaric acid when pH was constant (Zhao et al., 2023a).

With respect to the buffering capacity of wine, it is primarily determined by its organic acid content, especially tartaric acid, which is the main acid in wine and together with the concentration of other organic acids, such as malic, citric, succinic, lactic and acetic acids, as well as the ratio between malic and tartaric acid, play an essential role by acting in the form of wine salts, which serve as buffers, thus maintaining the pH of the wines in the range of 2.8–4.0. It has been reported that the buffering capacity is higher in wines with a pH close to the pKa of their main acidic components, being also higher in a hydroalcoholic solution (11% V/V) than in an aqueous solution (Obreque-Slier et al., 2016). In this sense, its positive influence on the prediction of SPI in this study could be mainly related to the concentration of different organic acids in the wine and/or with the ratio of their concentration.

Regarding the positive influence between the value at 520 nm and SPI prediction, it could be related to the well-known positive relationship of the 520 nm absorbance of anthocyanins to pH as a consequence of the total acidity of wines, with effects in anthocyanin-tannin polymerization and color stability (Sims and Morris, 1985).

In light of all these results, it is clear that the controversial issue of polymerization needs to be addressed, since neither the mDP nor the normalized mDP were selected in the variable selection step. In this regard, one of the possible explanations is that the variability of a large data set (real red wines) in concentration values of many parameters and their overall structure might obscured the specific effect of minor changes in mDP. Normally, as mentioned below, the effect of mDP is investigated in theoretical model systems lacking in this diversity, and this could be also the reason why alcohol concentration was not a selected parameter for the final model. Our model on the other hand, seeks for an overall practical investigation of the main factors common to most or all of the wine considered. However, it is important to mention the discordance found in the literature on the effect of mDP and wine astringency. In general terms, it has been stated that with an increase in the polymerization, and therefore the molecular weight, procyanidins increment their interaction with salivary proteins and, therefore, their precipitation and, consequently, the astringency (Ma et al., 2016). In fact, Pascotto et al. (2021), through asymmetrical flow field-flow fractionation coupled with multi-detection, found that only the fraction of wine polyphenols with a molecular weight <5 kDa is related with astringency sensation. However, there is no consensus on the actual effect of the mean degree of polymerization of real wine samples on perceived astringency. As procyanidin polymerization increases, the perception of astringency increases (Sun et al., 2013), whereas no significant correlation between mDP and astringency was found as well (Quijada-Morín et al., 2012). Indeed, Quijada-Morín et al. (2012) propose that for the conditions of their study, with mDP between 2.9 and 4.3, the astringency is more related to other factors such as procyanidin subunit concentration and composition than the degree of polymerization of procyanidins, as this present study may suggest. One answer to these contradictory results could lie in a magnitude-dependent effect of the mDP value. In this view, Kyraleou et al. (2016) found a positive effect of mDP on astringency but only for oligomers. Instead, Sáenz-Navajas et al. (2019) found that mDP effect on astringency could have a dynamic behavior from a threshold value of mDP 1.4 with an inverse correlation, thus decreasing astringency with increasing mDP. However, the mDP range for the latter study was only 0.1 to 2.8, falling outside the mDP values of the present study, which ranges (after treatment of outliers) from 8.8 to 29.7 with a mean of 15.6. However, Chira et al. (2009) found a positive effect mDP within a range between 4.3 and 48.8 and a mean of 21.4, but for skin grape extracts only and in one of the two vintages studied. The difficulty in simply relating the mDP value to the actual astringency of the wine sample may be due to the fact that its effect would be related to the hydrophobicity of the tannins. Larger condensed tannins contribute more hydrophobic groups that would increase tannin-protein interactions and, consequently, astringency. But, during tannin polymerization through wine maturation, conformational rearrangements and aggregation are thought to establish a non-linear relation between astringency and the increase of hydrophobic functional groups (Scollary et al., 2012; Sáenz-Navajas et al., 2019). Furthermore, these discordances in the literature on the structural effect of tannins on protein precipitation and/or astringency may indicate that the mechanism acting on the perception of astringency is not just the precipitation of salivary proteins by tannins. It could be important not only the amount of precipitated proteins and flavanols but also the remaining concentration in the supernatant of both proteins and flavanols, as it could also play a role in the perception of astringency (Kallithraka et al., 2001). These concepts are worth to be added in future works on predicting salivary protein precipitation or perceived astringency in order to have more robust prediction models and a better understanding of the complete mechanism behind astringency.

Finally, in reference to the robustness and weaknesses of the created PLSr model, although powerful statistical tests were used to ensure the significance of the model, it is necessary to remember that this is a preliminary attempt to predict and understand the phenomenon of human saliva precipitation in contact with wine from a real and a quasi-large data set.

## 5 Conclusions

The combined use of PLSr, VIP scores and jack-knifed regression coefficient *p*-value with Randomized Test is an effective and reliable data-driven model methodology for assessing a significative SPI prediction model using wine chemical variables when they are not strongly correlated (see Fig. S1). Due to the complex multivariate problem and the generation of a significant model for prediction, this approach is recommended for further research involving the elucidation of how a change in the composition of wine affects the perception of astringency. In particular, a big dataset of both samples and parameters (e.g., procyanidins with different structures and polymerization grades such as B1-8, C1-2, tetramers to pentamers, and further polymerizations), in conjunction with non-linear models and other systems of variable selection remains to be investigated to further develop the prediction model of astringency in wine. In this sense, some controversial issues could be addressed, such as (i) to model the effect of mDP procyanidin on salivary protein interaction and astringency as a function of a wide range of values; (ii) to elucidate how the structure of procyanidins has an effect on astringency dependent on variables such as %PC or mDP of them; (iii) to understand why the effect of the same variables on astringency changes in different vintages; (iv) to fit a model capable of compacting a small set of universal wine analytical variables able to predict astringency well in real samples of new wines with a wide concentration range of physico-chemical characteristics.

The results presented in this work are, to our knowledge, the first time that a large number of diverse red wines (110 mono-varietal samples produced from 11 grape cultivars) are analyzed to attempt to predict SPI from their chemical composition. This effort increases the understanding of the common physico-chemical variables related to the astringency phenomenon, as well as the management of a large database to predict future astringency-related issues, and through them, improving the ability of winemakers and wine researchers to produce wines with higher consumer liking with a holistic understanding of it.

## Funding

This work was supported by the Italian Ministero dell’Istruzione, Università e Ricerca (10.13039/501100003407MIUR) project PRIN 20157RN44Y.

## CRediT authorship contribution statement

**Cristian Galaz Torres:** Methodology, Validation, Formal analysis, Resources, Data curation, Writing – original draft, Writing – review & editing, Visualization, Conceptualization. **Arianna Ricci:** Methodology, Validation, Formal analysis, Writing – original draft, Conceptualization. **Giuseppina PaolaParpinello:** Methodology, Validation, Writing – original draft, Supervision, Conceptualization. **Angelita Gambuti:** Funding acquisition, Conceptualization, Methodology, Writing – review & editing. **Alessandra Rinaldi:** Funding acquisition, Conceptualization, Methodology, Formal analysis. **Luigi Moio:** Funding acquisition, Conceptualization. **Luca Rolle:** Funding acquisition, Conceptualization, Writing – review & editing. **Maria Alessandra Paissoni:** Funding acquisition, Conceptualization, Formal analysis. **Fulvio Mattivi:** Funding acquisition, Conceptualization, Writing – review & editing. **Daniele Perenzoni:** Funding acquisition, Conceptualization, Investigation. **Panagiotis Arapitsas:** Funding acquisition, Conceptualization, Investigation, Formal analysis, Data Curation. **Matteo Marangon:** Funding acquisition, Conceptualization, Writing – review & editing. **Christine Mayr Marangon:** Funding acquisition, Conceptualization, Writing – review & editing. **Davide Slaghenaufi:** Funding acquisition, Conceptualization, Writing – review & editing.**Maurizio Ugliano:** Funding acquisition, Conceptualization, Writing – review & editing. **Andrea Versari:** Methodology, Validation, Formal analysis, Resources, Data curation, Writing – original draft, Supervision, Project administration, Funding acquisition, Conceptualization.

## Declaration of competing interest

The authors declare that they have no known competing financial interests or personal relationships that could have appeared to influence the work reported in this paper.

## Data Availability

Specifications for winemaking protocol and sample storage, including wine analysis methodologies and the metadata of samples, are fully available in the literature from our previous cited works
