# Supplementary material for: Multivariate prediction of Saliva Precipitation Index for relating selected chemical parameters of red wines to the sensory perception of astringency
Source: Curr Res Food Sci. 2023 Oct 26;7:100626. doi: 10.1016/j.crfs.2023.100626 (PMC10651451; doi:10.1016/j.crfs.2023.100626)
Supplement: Multimedia component 1 [file mmc1.docx]

**Supplementary material.** Specifications of wine analysis methodologies for the red wines object of this study.

**Table S1.** List of the physicochemical variables that were used for this study together with the reference to the previous articles

where the methodologies can be found.

| Analysis | The method can be consulted at |
| --- | --- |
| pH | Giacosa *et al.*, 2021 |
| 230nm | Giacosa *et al.*, 2021 |
| 280nm | Giacosa *et al.*, 2021 |
| 230nm/280nm | Giacosa *et al.*, 2021 |
| 340nm | Giacosa *et al.*, 2021 |
| 420nm | Giacosa *et al.*, 2021 |
| 440nm | Giacosa *et al.*, 2021 |
| 520nm | Giacosa *et al.*, 2021 |
| 580nm | Giacosa *et al.*, 2021 |
| 620nm | Giacosa *et al.*, 2021 |
| Polymeric color (AU) | Giacosa *et al.*, 2021 |
| Copigmentation (AU) | Giacosa *et al.*, 2021 |
| Tannins reactive to iron (Tannins-Fe (mg/l)) | Giacosa *et al.*, 2021 |
| Phenolics reactive to iron (Phenolics-Fe (mg/l)) | Giacosa *et al.*, 2021 |
| small polymeric pigments (SPP (%)) | Giacosa *et al.*, 2021 |
| large polymeric pigments (LPP (%)) | Giacosa *et al.*, 2021 |
| Buffer capacity (meq/pH unit) | Giacosa *et al.*, 2021 |
| Total aldehydes (mg/l) | Giacosa *et al.*, 2021 |
| Antioxidant activity (ABTS (%)) | Giacosa *et al.*, 2021 |
| Tannin-Fe/Anthocyanin | Giacosa *et al.*, 2021 |
| Total anthocyanins index (mg malvidin-3-O-glucoside chloride/L) | Giacosa *et al.*, 2021 |
| Monomeric anthocyanins index (mg malvidin-3-O-glucoside chloride/L) | Giacosa *et al.*, 2021 |
| Total/monomeric anthocyanins | Giacosa *et al.*, 2021 |
| Total flavonoids index (mg (+)-catechin/L) | Giacosa *et al.*, 2021 |
| Total phenols (Folin-Ciocalteu) (mg (+)-catechin/L) | Giacosa *et al.*, 2021 |
| Proanthocyanidins assay (mg cyanidin chloride/L) | Giacosa *et al.*, 2021 |
| Vanillin assay (mg (+)-catechin/L) | Giacosa *et al.*, 2021 |
| Alcohol (% v/v) | Giacosa *et al.*, 2021 |
| Reducing sugars (g/L) | Giacosa *et al.*, 2021 |
| Titratable acidity (g tartaric acid/L) | Giacosa *et al.*, 2021 |
| Volatile acidity (g acetic acid/L) | Giacosa *et al.*, 2021 |
| Malic acid (g/L) | Giacosa *et al.*, 2021 |
| Lactic acid (g/L) | Giacosa *et al.*, 2021 |
| Tartaric acid (g/L) | Giacosa *et al.*, 2021 |
| Total dry extract (g/L) | Giacosa *et al.*, 2021 |
| Glycerol (g/L) | Giacosa *et al.*, 2021 |
| Protein content (mg/L) | Marangon *et al.*, 2022 |
| Polysaccharide content (mg/L) | Marangon *et al.*, 2022 |
| equivalent catechin (mg/L) | Arapitsas *et al.*, 2022 |
| Procyanidin B1 (mg/L) | Arapitsas *et al.*, 2022 |
| Procyanidin B2 (mg/L) | Arapitsas *et al.*, 2022 |
| Catechin (mg/L) | Arapitsas *et al.*, 2022 |
| Catechin terminal unit (mg/L) | Arapitsas *et al.*, 2022 |
| Epicatechin (mg/L) | Arapitsas *et al.*, 2022 |
| Epicatechin terminal unit (mg/L) | Arapitsas *et al.*, 2022 |
| Gallocatechin (mg/L) | Arapitsas *et al.*, 2022 |
| Gallocatechin terminal unit (mg/L) | Arapitsas *et al.*, 2022 |
| Epigallocatechin (mg/L) | Arapitsas *et al.*, 2022 |
| Epigallocatechin terminal unit (mg/L) | Arapitsas *et al.*, 2022 |
| Catechin gallate (mg/L) | Arapitsas *et al.*, 2022 |
| Catechin gallate terminal unit (mg/L) | Arapitsas *et al.*, 2022 |
| Catechin+epicatechin phloroglucinol (PHL) (mg/L) | Arapitsas *et al.*, 2022 |
| Epigallocatechin phloroglucinol (PHL) (mg/L) | Arapitsas *et al.*, 2022 |
| Epigallocatechin gallate phloroglucinol (PHL) (mg/L) | Arapitsas *et al.*, 2022 |
| Medium degree of polymerization (mDP) | Arapitsas *et al.*, 2022 |
| mDP normalized tannin-FE (mDP * tannin-FE/1000) | Calculated from Arapitsas *et al*., 2022 |
| Saliva Precipitation Index (SPI) (g/L GAE) | Rinaldi *et al.,* 2014 |


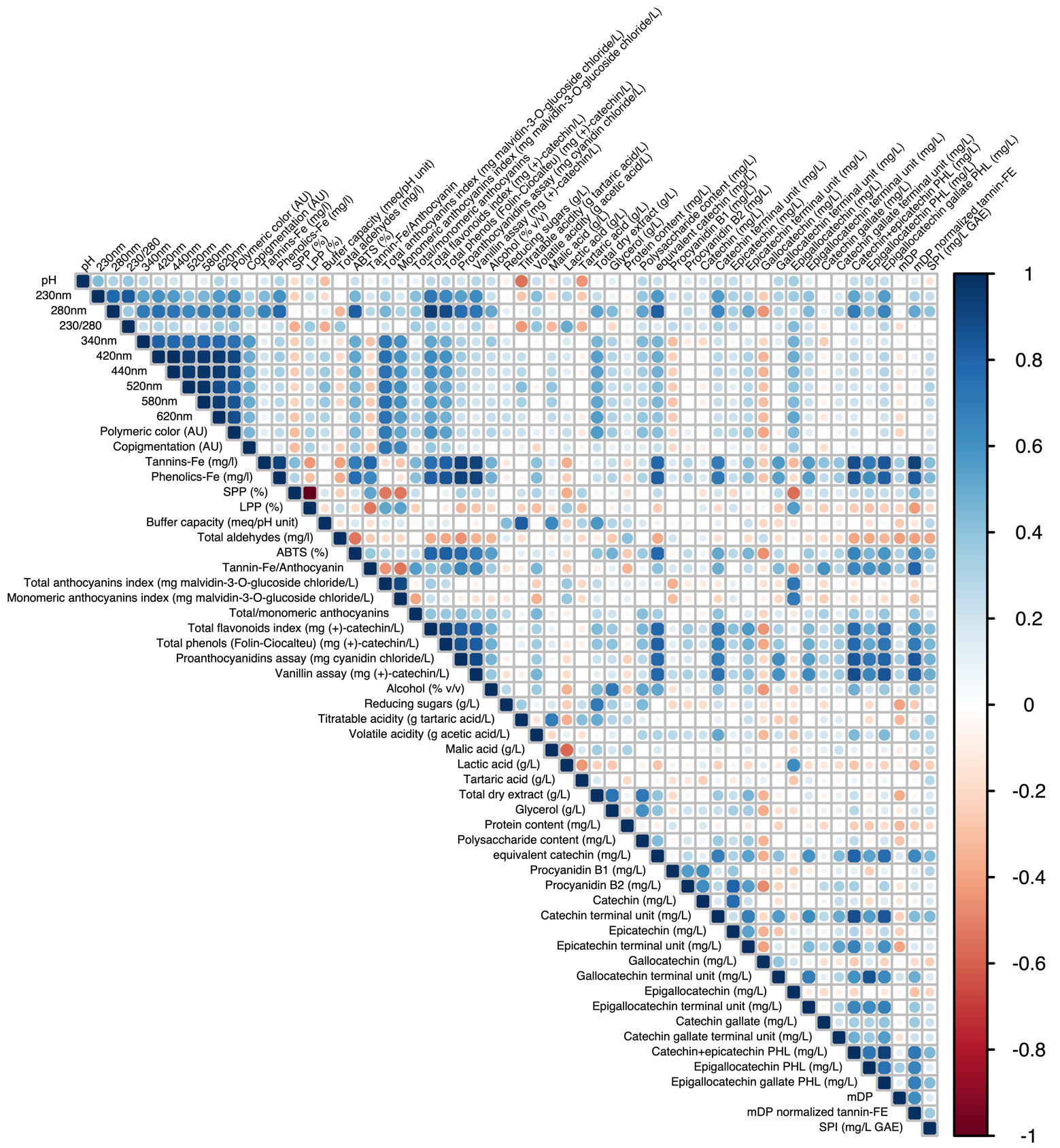


**Fig. S1.** Correlation plot (p-value: 0.05) original training data set.
